# Schick Test for Diphtheria Susceptibility

**Published:** 1916-07

**Authors:** 


**Schick Test for Diphtheria Susceptibility.** Don M. Griswold, Detroit, Jour. of Lab. & Clin. Med., Mich. Skin tests are von Pirquet's (first in point of time) for tuberculosis, and analogous tests as to the presence or absence of gonorrhoea, syphilis, and typhoid. The von Schick test is analogous but determines, not the presence of the disease but of susceptibility. Romer observed that persons having less than 1/30 unit of diphtheria antitoxin per C.C. of blood serum were susceptible to diphtheria and that nurses, internes and doctors (sic) frequently exposed and not contracting the disease, had more than this amount. Schick developed the technic of his test as follows: 1/50 M.L.D. diphtheria toxin, diluted to 1/10 C.C. injected into the skin is or is not neutralized according as the antitoxin present in the blood exceeds or falls below 1/30 unit of antitoxin. A chart is presented showing quite an accurate correspondence between the susceptibility as determined theoretically by the Schick test and the actual proportionate incidence of the disease. At the end of the first year, about 43% of Schick tests are positive, that is show susceptibility. At the end of the third year about 53% are positive. At the end of the 7th year, about 42%, at the end of the 9th, 32%, at the end of the 11th, 22%; at the end of the 13th, 12%. The proportionate distribution of diphtheria after the first two years, runs approximately 10% below the curve of susceptibility by the Schick test. (It should be noted that, for purposes of comparison, one should take not the percentage of incidence by age of actual diphtheria series, but the percentage of persons of a given age that contract the disease. In such a comparison, one would still expect that the susceptibility by the Schick test would run far above the incidence as, obviously, only a variable number of susceptible persons actually contract any disease, especially when it does not occur as an epidemic).

